# Helping each other to learn – a process evaluation of peer assisted learning

**DOI:** 10.1186/1472-6920-6-18

**Published:** 2006-03-08

**Authors:** Liam G Glynn, Anne MacFarlane, Maureen Kelly, Peter Cantillon, Andrew W Murphy

**Affiliations:** 1 Department of General Practice, Clinical Science Institute, National University of Ireland, Galway, Ireland

## Abstract

**Background:**

The benefits of Peer Assisted Learning (PAL) are well established with positive effects on examination scores, student satisfaction and personal and professional development reported. PAL is increasingly utilised as a resource within medical education where the restrictions on resources have forced teachers to look at creating new educational environments which can be delivered at a lower cost. This study sought to evaluate the *processes *at work as the emphasis of PAL research to date has largely been on the consideration of student outcomes.

**Methods:**

Fifth-year medical undergraduates, who had completed their communication skills modular training and attended a preparatory workshop, facilitated a role-play session for their second-year colleagues within an Early Patient Contact programme. Semi-structured interviews and focus groups were used to collect data at different time points in order to establish the views of peer learners and tutors towards this new method of teaching. The data was analysed according to the principles framework analysis using N-vivo software. Themes were shared and debated with the multidisciplinary team of authors and a concordance of views on common themes was reached after discussion and debate.

**Results:**

Analysis of the data resulted in the emergence of three thematic categories: Learning Environment, Educational Exchange and Communication and Modelling. The data demonstrated a concordance of the views between peer tutors and learners on barriers and levers of this approach as well as a heightened awareness of the learning environment and the educational exchange occurring therein.

**Conclusion:**

The data is significant as it not only demonstrates a high level of acceptability among tutors and learners for PAL but also indicates the reciprocity of educational exchange that appears to occur within the PAL setting. This study highlights some of the unique characteristics of PAL and we recommend the development of further qualitative studies around peer learners and tutors views of this process.

## Background

Peer assisted learning (PAL) has been defined as "the development of knowledge and skill through active help and support among status equals or matched companions" [1]. The same author has described the process of PAL as a situation in which "people from similar social groupings who are not professional teachers help each other to learn and learn themselves by teaching" [2]. It is this reciprocity of learning among other things that makes PAL such an attractive idea to educationalists. This is particularly true in medical education, where the restrictions on resources have forced teachers to look to creating new educational environments which can be delivered at a lower cost [3].

PAL has been embraced by medical educationalists for many years in the US [1,4,5]. In fact, the benefits of PAL have been extensively described in that part of the world and are reputed to positively correlate with examination performance [4,5]. The benefits associated with PAL, however, are not confined to examination scores. Other student benefits recognised are those of lowering subjective distress and enhancing course satisfaction through the establishment of a reciprocal social support system [6]. Researchers have also shown that pairing junior and senior undergraduate students provides psychological support and aids professional and personal development [7]. In addition to knowledge and skills mentioned above, modern learning objectives will often encompass "attitudes" as part of the learning process and this is particularly relevant in PAL as peers can prove powerful role models [8].

Additionally, the advantages of PAL do not appear to be limited just to the peer learners. Peer tutors also have been shown to benefit significantly in this learning environment [9]. In fact, in some studies it has been shown that peer tutors appear to show significantly greater cognitive gains than their peer learner counterparts [10,11].

The MB BCh BAO curriculum at National University of Ireland, Galway has a duration of six years. As part of this curriculum, the Department of General Practice runs an Early Patient Contact (EPC) programme over a six week period for second-year medical students (Table 1). This involves students visiting a local family doctor practice as well as a patient of that practice in their own home. During this visit they are asked to take a simple history of the patient's illness and examine the effect this illness has on the patient and their family. When the programme was originally piloted, feedback from the students indicated a lack of confidence in their ability to communicate effectively with the patients. The students suggested that a session on communication skills should be introduced into the course. A two hour session on communications skills based on the Calgary-Cambridge model [12] was designed to attempt to remedy this. The communication skills session incorporates basic communication skills theory as well a video demonstration of a fifth-year medical student communicating with a simulated patient. This is followed by small group role-play session (limited to six students). This experiential teaching method has been shown to be the most effective way of teaching communication skills [12]. These small group sessions have traditionally been facilitated by medical teachers from different disciplines, but faced with limited faculty resources, increased class sizes and difficulties in recruiting medical teachers we sought a solution to prevent the cancellation of this section of the communication skills curriculum.

The proposed solution was the introduction of PAL on a pilot basis for the academic year 2003-2004. We proposed that a group of fifth-year medical students would act as tutors for the small group role-play sessions. We felt it was important to fully evaluate this initiative as it was the first time that PAL had been used in our medical school. We sought to establish the views of learners and tutors towards this new method of teaching by conducting a process evaluation using qualitative methods. We were keen to evaluate the processes at work as the emphasis of PAL research to date has largely been on the consideration of student outcomes [13].

## Methods

**Figure 1:**
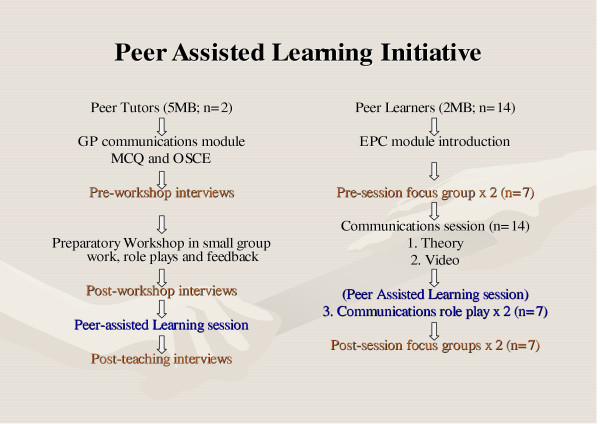
Peer Assisted Learning Initiative.

The PAL educational initiative is summarised in Figure 1.

### Peer Tutors (PT)

The role of the PT was to facilitate a small group experiential communication skills session using role-play. Tutors were selected from the fifth-year medical class. A short presentation about the PAL initiative was made to the class and volunteers were requested. Tutors were then chosen based on having successfully completed the following: communication skills and general practice modular training; summative assessment for that module which consisted of MCQ and OSCE examinations; all communication skills stations within the OSCE examination. Data were collected from the peer tutors in order to establish their expectations and views of PAL, using semi-structured interviews [14], at three time points:

*1. Prior *to delivering the session and prior to receiving any training.

*2. Following *a three hour preparatory workshop on how to run a small group, give feedback and conduct a role-play.

*3. Following *delivery of the role-play session to learners.

The framework for the topic guide for interviews is shown as Table 2. This was generated based on the study aims and objectives. In keeping with the iterative nature of qualitative research, it was modified during the data collection process in order to capture and revisit emerging themes as appropriate [15].

### Peer Learners (PL)

At the time of this study, peer learners were second-year medical students in the EPC programme who had volunteered to participate after a short presentation to their class. From the volunteer group, purposive sampling was used to select participants who reflected different gender and cultural backgrounds. The PL and PT group profile is described in Table 3. Data were collected from the PL to establish their expectations and views of PAL, using focus groups [16] at two time points:

*1. Prior *to the communication skills workshop with peer tutors.

*2. Following *the communication skills workshop.

The framework for the focus group topic guide is shown as Table 4. Its development and use was in line with the development and use of the interview topic guide described above. All interviews and focus groups were tape recorded with participants permission. Following verbatim transcription, data were analysed according to the principles of framework analysis [17] using Nvivo software [18]. Framework analysis involves the following five key stages:

#### i) Familiarisation

(preliminary examination of the data entailing an initial reading of all data)

#### ii) Developing a thematic framework

(producing analytical categories from respondents' statements or responses to the researchers enquiries and other key areas identified by respondents themselves)

#### iii) Indexing the material

(identifying instances of analytical categories involving searches for key words or phrases)

#### iv) Charting

(grouping instances under headings or particular research questions)

#### v) Mapping and interpretation to inform the key objectives of the research

(synthesising the range of views under particular themes to produce an overall picture on a range topics and relating this to other relevant research and theoretical perspectives)

Themes were shared and debated with the multidisciplinary team of authors which is known to heighten reflexivity in the interpretation process [19]. A concordance of views on common themes was reached after discussion and debate.

## Results

Analysis of the data resulted in the emergence of three thematic categories: Learning Environment, Educational Exchange and Communication and Modelling. A summary, descriptive account of the key findings from these categories follows. "Respondents were assigned a respondent code according to whether they were peer tutors (PT1 and PT2) or peer learners (PL), and whether the data was collected "pre" or "post" the PAL session. While every effort was made to systematically identify individual peer learners in the focus groups, poor quality of tape recording made it impossible to do so at the transcribing stage."

### Learning environment

Comments about the learning environment were positive. Both PL and PT described feeling more relaxed and comfortable during the session.

"I think it was a lot more informal and relaxed and it was like.... We weren't as, well I didn't feel as much pressure.." (PL) [post]

The importance of the learning environment was noted by tutors and learners. PT were aware of the idea of creating a "level playing field" (PT1) [pre] with "no barriers" (PT2) [pre]. Similarly, PL felt that the PT were "more down to earth" [post] and that they could "relate to them more" [post] than faculty tutors. This safeness described in the learning environment was recognised by PL prior to the workshop ....

"...you will feel more free to ask questions and gather information."

(PL) [pre]

...and appeared to enhance PL participation.

"I suppose you'd be more willing to ask questions of your peer off the top of your head whereas you might spend a whole lecture preparing your question."

(PL) [pre]

The safe learning environment seemed to be the basic building block that facilitated a growth of confidence in both learner and tutor. The fact that the PT were peers and had to ability to assimilate the material being taught and demonstrate it with confidence, made the PL feel that they would be able to achieve the same:

"I think even watching (the peer tutor)... there was a lot of things that he was using that he had learnt, and it's really encouraging to see that he was using them and he was well able to do it.." (PL) [post]

### Educational exchange

Once a degree of confidence and trust exists, it creates an ideal atmosphere for learning to take place. It was evident that this learning was a process of exchange and was recognised as such by both learners and tutors:

"When you teach something that's when you know it best"

(PL) [pre]

"I looked at it as an opportunity.....to better myself and.......to help others if I could and for them to help me"

(PT1) [post]

There was a feeling among the PL that the quality of the information provided by the PT would be of greater value as it was perceived to be more immediate, believable, relevant and useful.

"It might help (the peer tutors) as well because they're just after learning it so if they can pass on what they've learnt it'll help them to learn it, but eh, give us a different perspective of it as well"

(PL) [pre]

This was also recognised by the PT:

"It's always handy to have someone who's just done it and who has helpful hints as opposed to trying to figure them out for yourself"

(PT2) [pre]

Peer learners emphasised that they felt the PT would bring a unique and very useful perspective to the session due to their immediacy with the material and their student-like approach. They felt the PT would ensure that the session was relevant and would be aware of the potential learning difficulties and ways of surpassing these.

"It'll be a different perspective altogether when a senior tells you rather than a lecturer or a tutor...you might take it as a different approach and you might feel closer....you will feel more free to ask questions and gather information....you'll kind of feel ... well they'll have had the same problems as we have or the same difficulties." (PL) [pre]

This exchange of knowledge was also recognised as the area of greatest limitation for PAL generally. This was a concern for the PL who thought that...

"..one disadvantage of (peer learning) might be that the tutor might not have the same type of knowledge or experience as a (faculty member)"

(PL) [post]

...and for the PT who realised the limitations to their own knowledge...

"I think that's the biggest disadvantage for the students... that you wouldn't actually know it as well as an actual teacher."

(PT2) [post]

### Communication and modelling

It is evident from the data that communication and learning was taking place freely and on a number of different levels between tutors and learners.

"...if he was someone who was a tutor.... I think you'd spend so much time trying to get an intelligent question..... but with (the peer tutor) ..you just said what you thought anyway...... I think you'd be more likely to volunteer an answer with him rather than with a tutor..." (PL) [post]

This was especially pertinent as the PL were being taught communication skills and the PT became a useful role model in consciously and subconsciously demonstrating those very skills:

"...lot of things that we were told about this morning and that we had employed in the actual role play......eye contact, relaxed posture and making everyone around you feel relaxed....(the peer tutor) had been using them."

(PL) [post]

This concept of "modelling" extended beyond the material being taught however, and appeared to provide the PL with a newfound confidence in their ability to progress along the educational journey:

"....when I see him kinda being so down to earth and all the rest I can think well I can be that way as well...." (PL) [post]

## Discussion

The "safe" learning environment is perceived as a vital factor in effective teaching and learning. It is not surprising therefore, that this aspect of the PAL process appeared as a recurring theme in the data and was highlighted by both learners and tutors as a unique and fundamental tenant of this approach. Safe learning environments are vital in order to engage students in purposeful learning experiences, encourage constructive interactions among tutors and students and enable students to control their own learning effectively. The interactions that are fostered by the 'safe' learning environment in PAL appear to encourage learners to create associative links to their existing knowledge, to evaluate the truth of their emerging understandings and to elaborate the content of the lesson – all the while avoiding being seen to contravene the rules of order of a tutor-led lesson [20]. However it is also worth noting that concern has been expressed that the learning environment in PAL can in fact lead to a decline in the *quality *of student learning, such that it becomes less focussed on understanding course material than succeeding through a greater awareness of assessment demands [13]. This occurs as PAL helps students come to terms with the demands of their course but depending on the context this can be at the cost of understanding the deeper material they are studying [13]. Modification in peer tutor training to reduce the emphasis on strategic learning and instead promote the intrinsic benefits of PAL has been shown to be a way of averting this trend [21].

A sense of cooperation and closeness appears to permeate PAL as a whole and probably explains some of the reasons behind it's success. This is reported to be due to the concept of "promotive interaction". Promotive interaction describes how individuals encourage and facilitate each other's efforts in order to reach the groups goals [22]. This can be done by exchanging resources and information; giving and receiving feedback; and mutually influencing each other's reasoning and behaviour which is evident in the communication taking place and the 'modelling' effect noted in the data. Therefore, perhaps what PAL attempts to achieve is to make "the implicit nature of social learning explicit by encouraging active learning within social settings" [23]. In PAL the interaction between tutor and learner takes place on a number of different levels simultaneously. In this study, the PT uses the subject matter and techniques of the communication skills session to facilitate the teaching process. This not only provides a useful illustration of these techniques for the PL but also gives the PT an opportunity to practice the techniques during the session itself. So simultaneously, the PT communicates, tutors, learns and illustrates and in so doing provides, and is provided with, a very rich learning experience. It would seem that this correlates with the assertion of Parr and Townsend that there are peer effects in learning that originate in peer interactions and association [8]. It would appear that these "peer" effects in learning are multiple, complex and often occur simultaneously and in a reciprocal fashion [8].

There is also the suggestion that those involved in PAL learn not just about the material in question but also appear to learn about learning [24]. The transferable skills gained in this way enable heightened performance in areas of study other than those targeted by PAL such as the development of critical thinking skills [25]. Contrary to what one might think it is not just stronger students that can or should work as tutors. The benefits of working as a tutor in the PAL setting has been recognised in children [26] as well as adults [11,27]. In fact results in studies among children, suggest that serving as a tutor may be a particularly useful method for enhancing the academic performance of low-achieving children [26]. Annis et al compared the learning taking place between peer groups tutoring and being tutored and found that tutoring resulted in significantly greater content-specific and generalized cognitive gains than being tutored [11]. Peer tutoring thus appears to be a potentially powerful technique for increasing all levels of student learning. So why is that when we learn to teach we appear to learn better than when we learn to be tested. Benware [28] suggests when we learn to teach, we learn with a more active orientation. He described how students who learn to teach, are more intrinsically motivated, have higher conceptual learning scores, and perceive themselves to be more actively engaged with the environment than students who learn in order to be tested [28].

One of the chief concerns raised in this data by the PL was about the quality of the teaching. Indeed, this is probably one of the crucial distinctions that must be made in relation to PAL, in that the role of the PT should be one of facilitation rather than of teaching [29]. An interesting distinction was made between the large lecture setting in which learners felt the PT would lack credibility and knowledge; and small group work, which it was felt would ideally suit PAL. Indeed, there are obvious perils in making students teachers in that they are not qualified to be teachers, it is unfair to place such demands on them, they can give incorrect or misleading information and it detracts from what PAL is supposed to be about. Other concerns that have been noted in previous studies besides peer competence, are those of informed consent and accountability [30]. Additionally, it would appear that PAL does not suit all professional groups. For example, in a study using student nurses as teachers, it became apparent that the student nurses were uncomfortable with being used as tutors. They repeatedly questioned the intrinsic worth of this approach as a developmental tool, and considered the responsibility for teaching the content of parallel resource sessions to lie with nurse educators [31].

The small group sessions described in this study have traditionally been facilitated by medical teachers from different disciplines but were limited by lack of faculty resources, increased class sizes and difficulties in recruiting medical teachers. Peer tutoring can provide an innovative way of overcoming such difficulties. The cost of this approach is the provision of training for peer tutors. Although the issue of cost was not addressed in this study per se, the three hour preparatory workshop for prospective peer tutors, who can then go on to facilitate multiple role-play sessions in the place of medical teachers has the potential to be extremely cost-effective.

### Study limitations

This study does not include data on student outcomes after the introduction of the PAL approach and thus, we are not in a position to make comparative comments based on the empirical data collected. The purpose of this study was to evaluate the *processes *at work precisely because the emphasis of PAL research to date has largely been on the consideration of student outcomes. Despite the obvious importance of outcome measures in medical education research, there is a growing awareness that evaluation of any complex educational intervention such as PAL demands a complete programme of research not just a consideration of simple outcome measures [32]. This case report describes a closely supervised pilot study of a PAL initiative and therefore the generalisability of the data should not be exaggerated. However, the profile of the participants is similar to that of medical schools generally and the process of purposive sampling has ensured a mix of gender and cultural backgrounds.

It could be argued that the close supervision of the study could have generated a "Hawthorne" effect however the topic guide of the focus groups and semi-structured interviews ensured that leading questions were minimised. In addition, study results indicated both positive and more critical views of PAL and were, overall, consistent with other research in the area.

## Conclusion

By and large, healthcare and academic staff are enthusiastic about teaching, but are often limited by resources and staff numbers [33]. PAL is one way of answering these issues. This study has demonstrated a concordance of the views between peer tutors and learners on barriers and levers of this approach as well as a heightened awareness of the learning environment and the flow of communication therein. This is significant as it not only demonstrates a high level of acceptability among tutors and learners but also indicates the reciprocity of educational exchange that appears to occur within the PAL setting. Additionally, some of the learning experiences of tutor and learner appear to be unique to the PAL setting and so strengthen the argument for the formal inclusion of PAL in the curriculum. This study has highlighted some of the unique characteristics of peer assisted learning and we recommend the development of further qualitative studies around peer learners and tutors views of this process.

## Abbreviations

PAL – Peer Assisted learning

PT – Peer Tutor

PL – Peer learner

## Competing interests

The author(s) declare that they have no competing interests.

## Authors' contributions

LG coordinated the study, participated in the research design, the primary and confirmatory qualitative analysis and manuscript writing. AMacF conducted the interviews and focus groups, participated in the research design, the primary and confirmatory qualitative analysis and the manuscript writing. MK, PC and AM conceived the study, participated in the research design and commented on the manuscript. All authors read and approved the final manuscript.

## Funding

This study was funded by an Educational Innovations Fund grant awarded by the Centre for Excellence in Learning and Teaching, National University of Ireland, Galway, 2003/4.

## Pre-publication history

The pre-publication history for this paper can be accessed here:



## Figures and Tables

**Table 1:** Early Patient Contact Programme, 2003/4

**Week**	**Educational Event**	**Duration**
Week 1	Course introduction Communications skills session	1 Hour 2 Hours
Week 2	Visit to family practice	3 Hours
Week 3	Visit to patient	3 Hours
Week 4	Visit to family practice	3 Hours
Week 5	Visit to patient	3 Hours
Week 6	Presentation of case reports Course conclusion	2 Hours 1 Hour

**Table 2:** Interview topic guide for Peer Tutor semi-structured interviews

*Section I: "settling in" questions*
How are you feeling about the process so far?
Why do you think you're doing it?
What do you hope to gain from this experience?
Are you looking forward to it? (if not, why not?)
Do you think it will be helpful to you?
What do you hope to gain from this particular session?

*Section II: Knowledge of, and attitudes to peer learning*.
Are any of you familiar with the idea of peer learning in medical education?
Have any of you any previous experience of peer learning?

*Section III: Expectations of the peer learning session*
How/when did you find out about that this session would be peer learning?
What do you know about this peer learning project? Can you describe it to me?
What was your initial reaction?
What do you think the advantages/disadvantages might be for you? *(prompt: do you have any hopes or fears about the peer learning session?)*
Do you think the peer learning session will be any different from a standard teaching session? How? *(prompt: can you give me examples?)*
Overall, what do you hope to gain from the session?

**Table 3:** Age, Sex and Race profile of Peer Learner and Peer Tutor groups

**Peer Learner**	**Age**	**Sex**	**Race**
PL1	18	Female	Caucasian
PL2	19	Male	Asian
PL3	18	Female	Caucasian
PL4	25	Male	Caucasian
PL5	19	Female	Asian
PL6	18	Female	Asian
PL7	19	Male	Asian
PL8	21	Male	Black African
PL9	18	Female	Black African
PL10	20	Male	Asian
PL11	22	Male	Caucasian
PL12	19	Female	Asian
PL13	18	Female	Caucasian
PL14	19	Female	Caucasian
**Peer Tutor**	**Age**	**Sex**	**Race**
PT1	25	Male	Caucasian
PT2	23	Female	Caucasian

**Table 4:** Interview topic guide for Focus Groups

*Section I: "settling in" questions*
What do you think you'll cover in the early patient contact module?
Why do you think you're doing it?
What do you hope to gain from this module?
Are you looking forward to it? (if not, why not?)
Do you think it will be helpful to you when you are meeting patients?
What do you hope to gain from this particular session?

*Section II: Knowledge of, and attitudes to peer learning*.
Are any of you familiar with the idea of peer learning in medical education?
Have any of you any previous experience of peer learning?

*Section III: Expectations of the peer learning session*
How/when did you find out about that this session would be peer learning?
What do you know about this peer learning project? Can you describe it to me?
What was your initial reaction?
What do you think the advantages/disadvantages might be for you?
What do you think the advantages/disadvantages might be for the teacher? *(prompt: do you have any hopes or fears about the peer learning session?)*
Do you think the peer learning session will be any different from a standard teaching session? How? *(prompt: can you give me examples?)*
Overall, what do you hope to gain from the session?

## References

[B1] Topping KJ (1996). The effectiveness of peer tutoring in further and higher education: A typology and review of the literature. Higher Education (Historical Archive).

[B2] Topping KJ, Goodlad S (1998). The effectiveness of peer tutoring in further and higher education: A typology and review of the literature. Mentoring and Tutoring by Students.

[B3] Nestel D, Kidd J (2003). Peer tutoring in patient-centred interviewing skills: experience of a project for first-year students. Medical Teacher.

[B4] Trevino PM, Eiland DC (1980). Evaluation of basic science, peer tutorial programme for first- and second-year medical students. Journal of Medical Education.

[B5] Ebbert MR, Morgan PM, Harris LB (1999). A comprehensive student peer-teaching programme. Academic Medicine.

[B6] Fantuzzo JW, Dimeff LA, Fox SL (1989). Reciprocal peer tutoring: A multimodal assessment of effectiveness with college students. Teaching of Psychology.

[B7] Escovitz ES (1990). Using senior students as clinical skills teaching assistants. Academic Medicine: Journal Of The Association Of American Medical Colleges.

[B8] Parr JM, Townsend MAR (2002). Envoirnments, processes and mechanisms in peer learning. International Journal of Educational Research.

[B9] Fantuzzo JWRRECSDLA (1989). Effects of Reciprocal Peer Tutoring on Academic Achievement and Psychological Adjustment: A Component Analysis. Journal of Educational Psychology.

[B10] Lambiotte JGDDFHRH (1987). Manipulating cooperative scripts for teaching and learning. Journal of Educational Psychology.

[B11] Annis LF (1983). The processes and effects of peer tutoring. Human Learning: Journal of Practical Research & Applications.

[B12] Kurtz S, Silverman J, Draper J (2005). Teaching and Learning Communication Skills in Medicine..

[B13] Ashwin P (2003). Peer support: relations between the context, process and outcomes for students who are supported.. Instructional science.

[B14] Oppenheim AN (1992). Questionnaire design, interviewing and attitude measurement.

[B15] Greenhalgh T, Taylor R (1997). How to read a paper: Papers that go beyond numbers (qualitative research). BMJ.

[B16] Krueger RA, Casey MA (2000). Focus Groups.

[B17] Carter Y, Shaw S, Thomas C (1999). An introduction to qualitative methods for health professionals.

[B18] Richards L (2000). Using Nvivo in Qualitative Research.

[B19] Barry CABNBND (1999). Using reflexivity to optimise teamwork in qualitative research.. Qualitative Health Research.

[B20] Nuthall GA, Alton-Lee AG (1993). Predicting learning from student experience of teaching:A theory of student knowledge construction in the classroom.. American Educational Research Journal.

[B21] Packham G, Miller C (2000). Peer-Assisted Student Support: a new approach to learning. Journal of Further and Higher Education.

[B22] Johnson R, Johnson D (1989). Cooperation and competition theory and research.

[B23] McCarthy S, McMahon S, Hertz-Lazarowitz R and Miller N (1995). From convention to invention: three approaches to peer inetraction during writing.. Interaction in cooperative groups.

[B24] Price M, Rust C (1995). Laying firm foundations: The long-term benefits of supplemental instruction for students in large introductory courses.. Innovations in education and training international.

[B25] Koehler C (1995). Supplemental instruction and critical thinking. Supplemental instruction update.

[B26] Allen VL, Feldman RS (1973). Learning through tutoring: Low-achieving children as tutors. Journal of Experimental Education.

[B27] Bargh JA, Schul Y (1980). On the cognitive benefits of teaching. Journal of Educational Psychology.

[B28] Benware CA, Deci EL (1984). Quality of learning with an active versus passive motivational set. American Educational Research Journal.

[B29] Wallace J, Stephenson J (1997). Student as mentor and role model to support effective learning.. Mentoring - the new panacea?.

[B30] Greenwood CR, Carta JJ, Hall RV (1988). The use of peer tutoring strategies in classroom management and educational instruction. School Psychology Review.

[B31] Morris D, Turnbull P (2004). Using student nurses as teachers in inquiry-based learning. Journal of Advanced Nursing.

[B32] Schuwirth L, Cantillon P (2005). The need for outcome measures in medical education. BMJ.

[B33] Busari JOPKJSAJVDVCPEGG (2002). How residents perceive their teaching role in the clinical setting. Med Teach.

